# Maternal Morbidity Associated with Early Preterm Birth in Low-Risk Singleton Pregnancies

**DOI:** 10.3390/jcm13237061

**Published:** 2024-11-22

**Authors:** Moti Gulersen, Erez Lenchner, Alisha Goyal, Amos Grunebaum, Frank A. Chervenak, Eran Bornstein

**Affiliations:** 1 Division of Maternal-Fetal Medicine, Department of Obstetrics and Gynecology, Sidney Kimmel Medical College, Thomas Jefferson University, Philadelphia, PA 19107, USA; 2 Biostatistics and Data Management, New York University Rory Meyers College of Nursing, New York, NY 10010, USA; 3 Division of Maternal-Fetal Medicine, Department of Obstetrics and Gynecology, Lenox Hill Hospital—Zucker School of Medicine at Hofstra/Northwell, New York, NY 11549, USA

**Keywords:** maternal complications, severe maternal morbidity, blood transfusion, intensive care unit admission, extreme preterm, periviability

## Abstract

**Background/Objectives:** While neonatal morbidities associated with early preterm birth are known, the risks of maternal morbidities in these births remain unclear. Thus, we set out to assess the risk of maternal morbidities associated with early preterm births. **Methods:** Retrospective cohort study utilizing the United States (US) Natality Live Birth database from the Centers for Disease Control and Prevention (2016–2021). Low-risk singleton pregnancies were included. High-risk conditions such as out-of-hospital births, fetal anomalies, pregestational and gestational diabetes, and hypertensive disorders of pregnancy were excluded. The rates of several maternal morbidities were compared among three gestational age at birth groups: 23 0/7–27 6/7 (i.e., extreme preterm), 28 0/7–33 6/7 (i.e., early preterm), and 37 0/7–41 6/7 (i.e., term, reference group) weeks. Multivariable logistic regression was used to adjust outcomes for potential confounders. Data were presented as adjusted odds ratios (aORs) with a 95% confidence interval (CI). **Results:** 18,797,394 live births were analyzed. Extreme and early preterm birth were associated with increased odds of maternal transfusion (aOR 3.32, 95% CI 3.13–3.53 and aOR 2.96, 95% CI 2.86–3.07), uterine rupture (aOR 3.75, 95% CI 3.14–4.48 and aOR 4.13, 95% CI 3.76–4.54), unplanned hysterectomy (aOR 5.60, 95% CI 4.85–6.48 and aOR 5.92, 95% CI 5.47–6.40), and maternal admission to the intensive care unit (ICU, aOR 10.58, 95% CI 9.97–11.54 and aOR 10.13, 95% CI 9.77–10.50) compared to term birth. The odds of third- or fourth-degree perineal lacerations were decreased in both preterm birth groups compared to term birth. **Conclusions:** In addition to the known prematurity-related neonatal morbidities, extreme and early preterm births also impose a risk for maternal morbidities. Higher odds of maternal transfusion, uterine rupture, unplanned hysterectomy, and maternal admission to the ICU were detected in our cohort. These data should be taken into consideration when caring for patients with preterm births.

## 1. Introduction

Preterm birth complicates over 10% of pregnancies in the United States (US) and worldwide [1,2]. Among those, one-third are indicated due to maternal or fetal high-risk conditions and two-thirds occur following spontaneous preterm labor or preterm prelabor rupture of membranes (PPROM) [3]. A number of causal mechanisms for preterm birth have been described, including infection or inflammation, hemorrhage, uterine overdistension, stress, and other immunologically mediated processes [3]. The public health impact of preterm birth is well known; prematurity is responsible for both short- and long-term morbidities and is the leading cause of death in children under the age of 5 years globally [4,5]. Subsequently, the annual health care costs associated with preterm birth in the US has been estimated to be over 25 billion dollars [6].

While the increase in neonatal morbidities associated with early preterm birth have been well established [7], studies evaluating the accompanying risks of maternal morbidities, particularly in the absence of maternal medical indications for preterm delivery, are limited. This was, in fact, highlighted in a joint workshop by the National Institute of Child Health and Development, Society for Maternal–Fetal Medicine, American Academy of Pediatrics, and American College of Obstetricians and Gynecologists, identifying short- and long-term maternal morbidities associated with periviable birth as a key area for future research [8]. Cesarean deliveries are also more common in early preterm births, which are independently associated with an increased risk of maternal complications and have implications for future pregnancies [7,9,10].

The objective of this study was to determine the risk of several maternal morbidities associated with early preterm birth in low-risk singleton pregnancies.

## 2. Materials and Methods

This was a retrospective cohort study that utilized the US Department of Health and Human Services, Centers for Disease Control and Prevention (CDC), National Center for Health Statistics (NCHS), and Division of Vital Statistics Natality online database for the years 2016 to 2021. This database reports pregnancy and neonatal characteristics obtained from birth certificates of all live births in the US [11]. All singleton live births were eligible for inclusion. We excluded high-risk conditions such as out-of-hospital births, congenital anomalies, pregestational and gestational diabetes, chronic hypertension, and hypertensive disorders of pregnancy (including gestational hypertension and preeclampsia) to obtain our low-risk cohort. Deliveries with missing outcome data, late preterm (34 0/7–36 6/7 weeks of gestation), and post-term (≥42 weeks of gestation) births were also excluded.

Cases were stratified into three groups based on gestational age at delivery: 23 0/7–27 6/7 weeks (i.e., extreme preterm birth), 28 0/7–33 6/7 weeks (i.e., early preterm birth), and 37 0/7–41 6/7 weeks (i.e., term birth). Periviable births, defined as those occurring between 20 0/7–25 6/7 weeks, were not considered an independent group and instead included as a subset of extreme preterm births. The rates of several adverse maternal outcomes available in the database were compared between extreme and early preterm births to term births separately, with the term birth group serving as the reference group. These included maternal intensive care unit (ICU) admission, maternal blood transfusion, uterine rupture, unplanned hysterectomy, and third- or fourth-degree perineal laceration. By excluding medical comorbidities and pregnancy-related complications, we limited the potential impact of such maternal conditions or pregnancy complications to contribute to the maternal complications studied.

Baseline characteristics were compared among the three groups using Pearson’s chi-squared test with statistical significance set at *p* < 0.05. Comparisons of outcomes were made between extreme preterm births and early preterm births with term births separately. Multivariable logistic regression was performed to evaluate the association between each preterm birth group and adverse maternal outcomes, while adjusting for potential confounders such as maternal age, body mass index, race and ethnicity, tobacco use, chorioamnionitis (i.e., clinical chorioamnionitis or maternal temperature ≥ 38 degrees Celsius), and mode of delivery. Maternal race and ethnicity were self-identified on the birth certificate. Data were presented as adjusted odds ratios (aORs) with 95% confidence intervals (95% CI). All statistical analyses were performed using Stata Statistical Software 18.0 (StataCorp, College Station, TX, USA).

An institutional review board approval was not required, as the reported de-identified data are publicly available through a data use agreement with the NCHS.

## 3. Results

**Figure 1:**
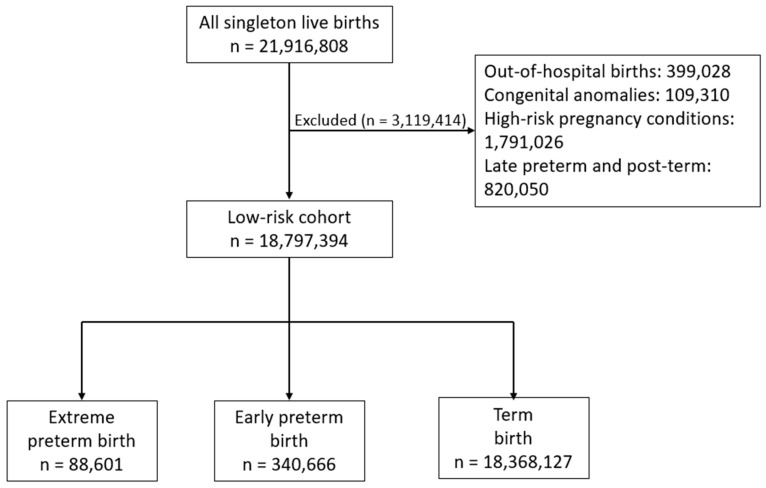
Flow diagram illustrating the exclusion process in the study cohort.

During the study period, there were a total of 18,797,394 low-risk live births eligible for inclusion. Of those, 88,601 (0.5%) were extreme preterm births (23 0/7–27 6/7 weeks), 340,666 (1.8%) were early preterm births (28 0/7–33 6/7 weeks), and 18,368,127 (97.7%) were term births (37 0/7–41 6/7 weeks). A detailed flowchart illustrating the exclusion process is presented in Figure 1. All baseline and pregnancy characteristics compared among the three groups were significantly different (Table 1). Patients in the extreme and early preterm birth groups had higher proportions of patients who were young or advanced maternal age, less educated, had higher BMI, were of non-Hispanic black race and ethnicity, used Medicaid insurance, and had no prenatal care compared to patients in the term birth group (Table 1). Additionally, there were higher rates of tobacco use, chorioamnionitis, and cesarean deliveries in extreme and early preterm births compared to term births (Table 1).

A comparison of the rates of adverse maternal outcomes compared separately between low-risk extreme and early preterm births with term births is displayed in Table 2 and Table 3. Extreme preterm birth was associated with significantly higher odds of maternal transfusion (aOR 3.32, 95% CI 3.13–3.53), uterine rupture (aOR 3.75, 95% CI 3.14–4.48), unplanned hysterectomy (aOR 5.60, 95% CI 4.85–6.48), and ICU admission (aOR 10.58, 95% CI 9.97–11.54) compared to term birth. The odds of third- of fourth-degree perineal laceration were significantly lower in extreme preterm births compared to term births (aOR 0.07, 95% 0.04–0.11).

Similar results were also identified when comparing adverse maternal outcomes in early term births versus term births. Early preterm birth was associated with significantly higher odds of maternal transfusion (aOR 2.96, 95% CI 2.86–3.07), uterine rupture (aOR 4.13, 95% CI 3.76–4.54), unplanned hysterectomy (aOR 5.92, 95% CI 5.47–6.40), and ICU admission (aOR 10.13, 95% CI 9.77–10.50) compared to term birth. The odds of third- or fourth-degree perineal laceration were significantly lower in early preterm births compared to term births (aOR 0.23, 95% 0.20–0.26).

## 4. Discussion

Our analysis, based on this large contemporary US cohort of low-risk singleton live births, detected higher odds of several maternal morbidities associated with both extreme and early preterm births compared to term births. Specifically, the odds of maternal transfusion, uterine rupture, unplanned hysterectomy, and maternal ICU admission were increased in extreme and early preterm births compared to term births. As expected, deliveries in both the extreme and early preterm periods were associated with lower odds of third- or fourth-degree perineal lacerations compared to that of term births.

Studies evaluating maternal morbidity associated with preterm birth have been overall scarce and limited to smaller cohorts with heterogenous study designs. Nevertheless, the few reported studies are consistent with our findings. The most comprehensive assessment to date was published by Rossi and DeFranco in a population-based cohort of 1,457,706 live births from Ohio between 2006–2015 [12]. Preterm birth cohorts evaluated in their study were initially stratified by those occurring between 20 0/6–25 6/7 and 26 0/6–36 6/7 weeks’ gestation with outcomes compared to term births. The rates of maternal transfusion (1.5% and 0.6%), uterine rupture (0.17% and 0.06%), unplanned hysterectomy (0.25% and 0.14%), and maternal ICU admission (0.95% and 0.45%) were significantly increased in both preterm birth groups compared to term births [12]. Furthermore, the risk of composite adverse maternal outcome associated with births stratified by gestational age of preterm delivery remained statistically significant when the authors excluded medical indications for delivery [12]. Our findings align with theirs and expand on them by including a contemporary cohort that represents all US births instead of a single State. Furthermore, we primarily focused on a low-risk population. In a secondary analysis of a US observational cohort including 2659 singleton preterm births between 23 0/7–33 6/7 weeks’ gestation, Reddy et al. reported that the risk of composite serious maternal complications (e.g., hemorrhage, infection, ICU admission) was significantly increased in preterm births occurring at earlier gestational ages (23–27 weeks) compared to later (28–31 or 32–33 weeks) [13]. The rates of ICU admission were similar among the three preterm birth groups and higher overall (3.3%) compared to preterm births in our study (1.7%). This was likely secondary to the inclusion of a large proportion of patients with hypertensive disorders (36.6%) in their study [13], whereas we excluded high-risk conditions to assess the impact of early gestational age on the likelihood of maternal complications. While the rates of hemorrhage, a composite outcome including either blood loss ≥ 1500 mL, transfusion, or hysterectomy for hemorrhage was significantly increased in their cohort of preterm births occurring at earlier gestational ages, data regarding blood transfusion specifically were not reported [13].

Our findings that extreme and early preterm birth were associated with increased maternal morbidity in the absence of medical comorbid conditions indicating delivery highlight the importance of clinicians’ awareness that preterm birth is not only a major cause for neonatal morbidity but can also be associated with maternal morbidity. This should be incorporated in patient counseling when caring for pregnancies at risk of preterm birth. Furthermore, obstetric providers should be aware of these complications, ensure patients are being cared for in the appropriate settings, and incorporate management strategies to help reduce the incidence of such risks, as well as backup systems to allow for appropriate management of these complications. Several mechanisms may have contributed to our findings. Preterm birth is associated with higher rates of retained placenta, which is a risk factor for postpartum hemorrhage [14,15]. Patients with cervical insufficiency or PPROM often have subclinical signs of infection with higher circulating levels of inflammatory markers and cytokines, which may lead to an increased risk of complications at the time of delivery [16,17,18,19]. The association between extreme and early preterm birth and lower odds of third- and fourth-degree perineal laceration was not surprising. Given that birthweight is considered a major risk factor for advanced perineal lacerations [20], the lower birthweight in preterm births compared to term births likely contributed to this finding.

This study has several strengths. We address an important and understudied topic, exploring the association between early preterm birth and maternal morbidity, outlined by several professional organizations as a key area for future research. We utilized a contemporary, large, and comprehensive national database including over 18.5 million live births with significant power to detect differences in the outcomes evaluated. The use of the entire US birth registry makes our findings generalizable. Finally, a major strength is that we excluded patients with independent risk factors for maternal morbidity, such as comorbid maternal conditions and pregnancy-related complications, focusing on a low-risk population of preterm and term births.

This study also has several limitations, most of which are related to the inherent retrospective nature of birth certificate databases, variation in quality and accuracy of reporting among different institutions included, and information available for analysis. Although adjusted for in our analysis, the rates of chorioamnionitis and cesarean delivery were significantly higher in the extreme and early preterm births compared to term. Previous studies examining adverse maternal outcomes associated with chorioamnionitis and cesarean delivery at early gestational ages have demonstrated significantly higher odds of maternal morbidities such as transfusion and ICU admission [9,21]. Earlier cesarean delivery is associated with a higher likelihood of a vertical hysterotomy, which further increases the risk of perioperative morbidities [22]. Data regarding uterine incision type for those who underwent a cesarean delivery were not available in the database. In addition, data regarding antenatal interventions intended to delay delivery or improve neonatal outcomes that may impact maternal outcomes such as physical exam-indicated cerclage placement were unknown. Lastly, we were unable to exclude all possible medical indications for delivery, and it is possible that unmeasured confounders may have contributed to our findings.

In conclusion, our findings illustrate that extreme and early preterm birth are not solely a neonatal concern. These preterm births are also associated with increased odds of maternal complications such as transfusion, uterine rupture, unplanned hysterectomy, and maternal admission to the ICU. These data should be taken into consideration by the obstetrical team in addition to the associated neonatal risks. This can be incorporated into patient counseling, as well as used to ensure the appropriate setting and expertise to attempt prevention, early detection, and management of such maternal complications. Future research should focus on implementing institutional guidelines for management of such patients in order to reduce the risk of maternal morbidity.

## Figures and Tables

**Table 1:** Baseline and pregnancy characteristics compared among the three groups.

	Extreme Preterm Births 23 0/7–27 6/7 Weeks (*n* = 88,601)	Early Preterm Births 28 0/7–33 6/7 Weeks (*n* = 340,666)	Term Births 37 0/7–41 6/7 Weeks (*n* = 18,368,127)	*p* Value
Maternal age (years)				<0.001
<20	6179 (7.0)	19,405 (5.7)	880,428 (4.8)	
20–24	18,106 (20.5)	64,808 (19.1)	3,549,418 (19.4)	
25–29	23,447 (26.6)	88,662 (26.1)	5,310,755 (29.0)	
30–34	22,621 (25.6)	92,045 (27.1)	5,307,570 (29.0)	
35–39	14,159 (16.0)	58,089 (17.1)	2,715,975 (14.8)	
≥40	3752 (4.3)	16,098 (4.7)	564,337 (3.1)	
Body mass index (kg/m^2^)				<0.001
<18.5	2842 (3.4)	12,811 (3.9)	556,879 (3.1)	
18.5–24.9	27,035 (32.4)	116,820 (35.9)	7,506,115 (41.7)	
25–29.9	21,021 (25.2)	82,078 (25.2)	4,830,199 (26.9)	
30–34.9	15,627 (18.7)	56,152 (17.3)	2,759,017 (15.3)	
35–39.9	9290 (11.1)	31,386 (9.6)	1,360,927 (7.6)	
≥40	7619 (9.1)	26,050 (8.0)	970,686 (5.4)	
Education				<0.001
Less than high school	13,530 (15.7)	52,621 (15.7)	2,231,357 (12.3)	
High school graduate	28,002 (32.4)	100,989 (30.2)	4,723,452 (26.0)	
Some college credit	26,645 (30.8)	99,780 (29.9)	5,118,070 (28.2)	
College graduate	11,968 (13.8)	51,325 (15.4)	3,789,271 (20.9)	
Master’s or higher	6287 (7.3)	29,550 (8.8)	2,270,181 (12.5)	
Insurance type				<0.001
Medicaid	46,288 (52.2)	172,316 (50.6)	7,739,174 (42.1)	
Private	34,652 (39.1)	140,657 (41.3)	9,239,734 (50.3)	
Self pay/other	7661 (8.6)	27,693 (8.1)	1,389,219 (7.6)	
Race or ethnic group				<0.001
Non-Hispanic white	30,094 (34.0)	143,087 (42.0)	9,368,005 (51.0)	
Non-Hispanic black	29,407 (33.2)	83,476 (24.5)	2,574,516 (14.0)	
Other/multiracial	3929 (3.7)	14,952 (4.4)	697,087 (3.8)	
Asian and Pacific Islander	4271 (5.6)	19,081 (5.6)	1,250,744 (6.8)	
Hispanic	20,830 (23.5)	80,070 (23.5)	4,477,775 (24.4)	
Prenatal Care				<0.001
Initiated in first-trimester	60,823 (74.4)	238,784 (74.3)	14,070,178 (78.3)	
Initiated in second-trimester	13,061 (16.0)	51,775 (16.1)	2,844,413 (15.8)	
Initiated in third-trimester	55 (0.1)	8592 (2.7)	792,373 (4.4)	
No prenatal care	7822 (9.6)	22,281 (6.9)	263,657 (1.5)	
Tobacco use during pregnancy	7841 (8.9)	33,009 (9.8)	1,097,338 (6.0)	<0.001
Chorioamnionitis	4544 (5.4)	7746 (2.4)	280,394 (1.6)	<0.001
Cesarean delivery	57,367 (64.7)	204,687 (60.1)	5,490,890 (29.9)	<0.001

Data are presented as number (percentage). Pearson’s chi-squared test was utilized for comparison.

**Table 2:** Adverse maternal outcomes compared between extreme preterm births and term births.

	Extreme Preterm Births 23 0/7–27 6/7 Weeks (*n* = 88,601)	Term Births 37 0/7–41 6/7 Weeks (*n* = 18,368,127)	Unadjusted OR (95% CI)	* Adjusted OR (95% CI)
Maternal transfusion	1457 (1.6)	59,316 (0.3)	5.16 (4.90–5.44)	3.32 (3.13–3.53)
Uterine rupture	181 (0.2)	4783 (0.03)	7.86 (6.78–9.12)	3.75 (3.14–4.48)
Unplanned hysterectomy	245 (0.3)	5451 (0.03)	9.34 (8.22–10.62)	5.60 (4.85–6.48)
ICU admission	1731 (2.0)	17,507 (0.1)	20.89 (19.88–21.96)	10.58 (9.97–11.54)
Third- or fourth-degree perineal laceration	25 (0.03)	146,849 (0.8)	0.04 (0.02–0.05)	0.07 (0.04–0.11)

* Models adjusted for maternal age, body mass index, race and ethnicity, tobacco use, chorioamnionitis, and mode of delivery.

**Table 3:** Adverse maternal outcomes compared between early preterm births and term births.

	Early Preterm Births 28 0/7–33 6/7 Weeks (*n* = 340,666)	Term Births 37 0/7–41 6/7 Weeks (*n* = 18,368,127)	Unadjusted OR (95% CI)	* Adjusted OR (95% CI)
Maternal transfusion	4483 (1.3)	59,316 (0.3)	4.12 (3.99–4.24)	2.96 (2.86–3.07)
Uterine rupture	601 (0.2)	4783 (0.03)	6.79 (6.23–7.39)	4.13 (3.76–4.54)
Unplanned hysterectomy	970 (0.3)	5451 (0.03)	9.62 (8.99–10.30)	5.92 (5.47–6.40)
ICU admission	5620 (1.7)	17,507 (0.1)	17.59 (17.06–18.13)	10.13 (9.77–10.50)
Third- or fourth-degree perineal laceration	336 (0.1)	146,849 (0.8)	0.12 (0.11–0.14)	0.23 (0.20–0.26)

* Models adjusted for maternal age, body mass index, race and ethnicity, tobacco use, chorioamnionitis, and mode of delivery.

## Data Availability

These data are publicly available.
